# Validation of the Musculoskeletal Health Questionnaire in a general population sample: a cross-sectional online survey in Hungary

**DOI:** 10.1186/s12891-022-05716-9

**Published:** 2022-08-13

**Authors:** Áron Hölgyesi, Gyula Poór, Petra Baji, Zsombor Zrubka, Miklós Farkas, Ágota Dobos, László Gulácsi, Levente Kovács, Márta Péntek

**Affiliations:** 1 Doctoral School of Molecular Medicine, Semmelweis University, Budapest, Hungary; 2 Department of Health Technology Assessment, National Institute of Pharmacy and Nutrition, Budapest, Hungary; 3 Department of Rheumatology, National Institute of Rheumatology and Physiotherapy, Budapest, Hungary; 4 Department of Health Economics, Corvinus University of Budapest, Budapest, Hungary; 5 Health Economics Research Center, University Research and Innovation Center, Óbuda University, Budapest, Hungary; 6 Corvinus Institute for Advanced Studies, Corvinus University of Budapest, Budapest, Hungary; 7 Department of Accounting and Finance, University of Bristol, Bristol, UK; 8 Corvinus Center for Foreign Language Education and Research, Corvinus University of Budapest, Budapest, Hungary; 9 Physiological Controls Research Center, University Research and Innovation Center, Óbuda University, Budapest, Hungary

**Keywords:** Musculoskeletal health, Quality of life, Well-being, Patient reported outcome measures, Validation, Psychometrics, Population sample, Hungary

## Abstract

**Background:**

The Versus Arthritis Musculoskeletal Health Questionnaire (MSK-HQ) measures symptom severity and health-related quality of life (HRQoL) of people with musculoskeletal (MSK) conditions. We aimed to test the psychometric properties of the MSK-HQ among the general adult population and identify the determinants of MSK-HQ states. In addition, we aimed to explore the relationship between MSK-HQ and standard well-being measurement tools.

**Methods:**

The translation proccess of the MSK-HQ into Hungarian followed the standard methods provided by the developer. A cross-sectional online survey was performed in Hungary involving a population normative sample (*N* = 2004, women: 53.1%; mean age: 48.3, SD = 16.6 years). Socio-demographic characteristics and self-reported MSK disorders were recorded. Alongside the MSK-HQ, standard measures of HRQoL (EQ-5D-5L), physical functioning (HAQ-DI) and well-being (ICECAP-A/O, WHO-5, Happiness VAS) were applied. Clinical and convergent validity were assessed by subgroup comparisons (Mann–Whitney-U and Kruskal–Wallis tests) and Spearman’s rank correlations. Internal consistency was assessed by Cronbach’s alpha. Test–retest reliability (*N* = 50) was evaluated by intraclass correlation coefficient (ICC). Predictors of MSK-HQ were analysed by ordinary least square multiple regressions.

**Results:**

The mean MSK-HQ index score was 44.1 (SD = 9.9). MSK-HQ scores were significantly lower in subgroups with self-reported MSK disorders. Correlations were strong between MSK-HQ and EQ-5D-5L (0.788), EQ VAS (0.644) and HAQ-DI (-0.698) and moderate with the well-being measures (*p* < 0.05). Cronbach’s alpha was 0.924 and ICC was 0.936 (*p* < 0.05). Being a man, living in the capital, having higher income and education were positively associated with MSK-HQ scores.

**Conclusions:**

This is the first study to prove the validity and reliability of the MSK-HQ among the general public. The impact of socio-demographic characteristics on MSK-HQ scores deserves consideration in clinical studies.

**Supplementary Information:**

The online version contains supplementary material available at 10.1186/s12891-022-05716-9.

## Plain English summary

Musculoskeletal (MSK) disorders are among the most common chronic diseases affecting approximately 1.71 billion people worldwide and one in four people in Europe with an increasing prevalence by age. The Versus Arthritis Musculoskeletal Health Questionnaire (MSK-HQ) was developed to assess symptoms and quality of life of people with diverse MSK conditions (such as arthritis or back pain) in a standardised and comparable way. The measurement properties of the MSK-HQ have been proven in various MSK patient samples. However, little is known about its performance among the general public and how differences in socio-demographic status (e.g. age, sex, residency, educational level) of the respondents may influence the results. In our study we addressed these two main issues. We have produced and validated the Hungarian version of the MSK-HQ in an online survey involving a representative sample of the adult Hungarian population. Results of the MSK-HQ were compared against respondents’ health status, MSK problems and standard measures of health-related quality of life, physical functioning and wellbeing. Statistical analyses followed international standards. Results confirmed that MSK-HQ is a credible measurement tool for the assessment of MSK health-related quality of life of the general public. Certain socio-demographic factors (sex, residency, educational and income level) were shown to have significant impact on MSK-HQ results that should be considered in both the design of and conclusions drawn from MSK-HQ clinical studies.

## Background

Musculoskeletal (MSK) disorders are among the most common chronic diseases world wide that may have a negative impact on patients’ physical functioning, health-related quality of life (HRQoL) and ability to work, causing significant social and economic burden [1, 2].

The increasing use of generic HRQoL measures (such as the EQ-5D-5L, SF-36) can be observed in MSK diseases both in clinical trials and practice, however these tools do not specifically capture MSK health-related problems [3]. There has therefore been demand for an instrument which focuses on the impact of MSK disorders on patients’ life that can be used across different MSK conditions thus allowing for the comprehensive assessment of overall MSK health [4–7]. The Versus Arthritis Musculoskeletal Health Questionnaire (MSK-HQ) has been developed with the intent to fill this gap as it incorporates the assessment of patients’ MSK related symptoms and HRQoL aspects in one questionnaire and can be used in various MSK conditions [8]. The MSK-HQ also enables monitoring patients with MSK problems over time and makes it possible to compare the performance of health services utilized due to MSK disorders [8–10]. The MSK-HQ has been validated in selected patient samples with a wide range of MSK diagnosis and a number of language versions have become available as well [8, 9, 11–18]. However, the MSK-HQ has not been tested in the general population so far, therefore population normative data are not available and little is known about the impact of socio-demographic factors on MSK-HQ results.

In addition, most MSK-HQ validation studies applied only the EQ-5D-5L generic HRQoL measure and disease-specific tools as reference standards. The relationship between MSK-HQ and well-being measures has remained unexplored so far, while the assessment of the impact on well-being is receiving increasing attention in medical decision making [19, 20], health economic evaluations [21, 22], policy making [23] and in developing social care interventions [24]. The validation of the Hungarian language version would allow us to contribute to MSK-HQ research by targeting these questions, and also to broaden the use of the MSK-HQ instrument to Hungary.

The focus of this study is two-fold. Primarily, we aimed to establish and validate the Hungarian version of the MSK-HQ, analyse its psychometric properties among the general population and evaluate the effects of socio-demographic factors on MSK-HQ outcomes. Secondarily, we aimed to assess the associations between MSK-HQ and commonly used well-being measures.

## Methods

### Data collection

A cross-sectional online survey was conducted in May–June 2020 among the general adult population (age ≥ 18) of Hungary. Recruitment of respondents and data collection were performed by a survey company (New Land Media Kft. – Századvég) involving members of an online access panel. Details on participation rate were confidential. The company was responsible also for data protection and compliance with GDPR. Anonymized data were handled for the analyses. Quota sampling (a non-probability sampling method) was used in order to obtain a representative sample of the normal adult population. Quotas were set for age, sex, educational level and type of settlement according to the 2011 Population Census [25]. The targeted sample size was 2000, which is the double of the usually needed sample size (*N* = 1000) to achieve representativeness defined by the above detailed criteria. Nonetheless, we aimed to achieve a larger than a minimum sample size in all age-groups and a remarkable number of participants with MSK problems. Ethical approval was obtained from the Hungarian Medical Research Council (no. IV/565–5/2020/EKU). Respondents were informed that participation in the survey was voluntary, their data would remain anonymous, impersonal and would be used solely for scientific purposes. Before starting the survey, informed consent was obtained from all individual participants included in the study.

### The questionnaire

The survey questionnaire consisted of two modules. The first module (reported in this paper) focused on MSK health, the second one assessed respondents’ subjective expectations towards the length and quality of their life. Socio-demographic characteristics of the sample was recorded. Participants completed the Hungarian version of MSK-HQ along with standard outcome measures of health status, HRQoL, physical functioning and well-being. MSK health problems and diagnoses were explored with relevant questions of the European Health Interview Survey (EHIS) [22]. Respondents were asked to indicate if they used healthcare services (hospital admission, specialist care, general practitioner) or received informal care due to MSK problems. Informal caregivers were also identified by self-reports regardless of MSK health status. To assess test–retest reliability, the completion of MSK-HQ was repeated in the end of the survey in a subgroup of 50 respondents who were selected randomly from the study sample.

### Outcome measures

#### MSK-HQ and its translation and validation for Hungary

The MSK-HQ was developed in 2016 as a specific outcome measure to be used by people with different MSK conditions [8]. The questionnaire assesses how much respondents’ MSK problems have affected their life in the last two weeks. It consists of 14 items, from which eleven measure symptoms and HRQoL, while three focus on patients’ attitude towards their condition and the overall impact of symptoms. Response options are operationalized on a five-point Likert scale, ranging from 4 to 0 (ʻnot at all’ and ʻextremely’, respectively), except items 12 and 13, which are given in reverse order. The final score, ranging from 0 to 56, is calculated by summing up the scores given for each item. Higher scores indicate better MSK health status. An additional, fifteenth item assesses the number of days with significant physical activity in the past week but this item is not included in the final score.

The validation of the Hungarian version was carried out in accordance with the protocol provided by the developer of the original MSK-HQ (Oxford University Innovation). In brief, forward translations were performed independently by three researchers. A reconciled Hungarian version was produced via discussions which was then back translated independently by two experts. Results were reviewed by the developer and items with uncertanities went through a second round forward-back translation cycle. Cognitive debriefing was performed involving five patients with diverse MSK problems. After proofreading and quality check, the final Hungarian version was accepted as the best available language version by the developer.

#### MEHM

The Minimum European Health Module (MEHM) is composed of three questions concerning three different aspects of health: self-percieved health (response options: very good, good, fair, bad, very bad), long standing illness or chronic morbidity (response options: yes or no) and the presence of long-standing activity limitations measured by the Global Activity Limitation Indicator (GALI) (reponse options: severely limited, limited but not severely, not limited at all) [26].

#### EQ-5D-5L

The EQ-5D-5L is a generic questionnaire that evaluates HRQoL by assessing the following five domains: mobility, self-care, usual activities, pain/discomfort, anxiety/depression [27]. Respondents are asked to indicate their current health state on a five-level Likert-scale (ranging from 1 –’no problems’ to 5 –’unable to’). To calculate EQ-5D-5L index score, we used the Hungarian tariffs (range: -0.848 – 1.000) [28]. EQ VAS is an additional item that measures respondents self-reported health on a visual analogue scale, ranging from 0 to 100, where 0 indicates the worst and 100 indicates the best health status that the respondent can imagine. Higher scores indicate better health status.

#### HAQ-DI

Health Assessment Questionnaire – Disability Index (HAQ-DI) [29] measures functional ability over the past week with 20 items across the following 8 domains: dressing, arising, eating, walking, hygiene, reach, grip and common activities. Each domain contains 2 or 3 items. Possible response options for each item range from 0 to 3 (0—ʻwithout difficulty’ and 3—ʻunable to do’, respectively). Eighteen additional items are provided to indicate if aids or assisstive devices were used for the activities listed in the domains. To calculate the HAQ-DI score, we applied the alternative scoring method (no correction for aid or devices was made) thus the highest item score within each domain was considered to calculate the total score (range 0–3, higher score indicates worse status).

#### ICECAP-A and ICECAP-O

The ICECAP-A and ICECAP-O tools were developed to evaluate capability well-being of adults (18 +) and older people (65 +), respectively [30, 31]. Each version contains five items. For ICECAP-A these are attachment, stability, achievement, enjoyment and autonomy, while ICECAP-O items are attachment, security, role, enjoyment and control. Respondents are asked to indicate their current capability well-being on a 4-level scale (ranging from 4—ʻfull capability’ to 1—ʻno capability’). Index scores for each state can be calculated by using tariffs obtained from population level valuation studies. We used the validated Hungarian version of the questionnaire [32]. At the time of our analysis tariffs were available only for the United Kingdom so we used those for the calculations [31, 33].

#### WHO-5

The World Health Organisation-Five Well-Being Index (WHO-5) evaluates self-reported mental well-being in relation to the last two weeks [34]. The questionnaire comprises five items. Response options are available on a six-level scale (ranging from 0—ʻat no time’ to 5—ʻall of the time’). The final score is calculated by the multiplication of the sum of item scores by 4. Higher scores indicate better well-being.

#### Happiness VAS

A visual analogue scale (VAS) of happiness was applied to measure the current subjective degree of happiness of respondents (where 0 indicates ʻcompletely unhappy’ and 10 indicates ʻcompletely happy’).

### Statistical analysis

Socio-demographic characteristics and health status of the sample were analyzed using descriptive statistics. The psychometric characteristics of MSK-HQ were assessed in relation to socio-demographic characteristics, self-reported MSK status and diagnosis, healthcare and informal care utilisation, general health status (MEHM), HRQoL (EQ-5D-5L), physical functioning (HAQ-DI) and well-being (ICECAP-A/-O, WHO-5, Happiness VAS). The COnsensus-based Standards for the selection of health status Measurement INstruments (COSMIN) was applied so that we investigated construct validity (clinical, convergent, discriminant validity), internal consistency and reliability [35].

#### Validity

To assess clinical validity, we investigated whether the MSK-HQ can differentiate between subgroups using Mann–Whitney-U and Kruskal–Wallis tests for comparisons. The association between MSK-HQ score and socio-demographic characteristics were further investigated using ordinary least square (OLS) multiple regression analysis.

To evaluate convergent validity, Spearman’s correlation was calculated between MSK-HQ, EQ-5D-5L, EQ VAS, HAQ-DI, ICECAP-A/-O, WHO-5 and Happiness VAS scores. We also investigated correlations between MSK-HQ items, EQ-5D-5L domains and HAQ-DI domains. We expected strong correlation with the ʻMobility’ and ʻPain/discomfort’ domains of the EQ-5D-5L. The correlations were considered to be strong if the coefficient was over 0.5, moderate between 0.5 and 0.3, and weak under 0.3 [36]. In the multiple regression analysis we also explored associations between MSK-HQ, HRQoL (EQ-5D-5L) and functional status (HAQ-DI) controlling for socio-demographic characteristics. Three models were developed. In the first one, we included the EQ-5D-5L score, in the second one, the HAQ-DI, and in the third model, we included both the EQ-5D-5L and HAQ-DI score to examine how these two scales influence each other’s effect. All models were controlled for basic sociodemographic variables (sex, age, education, residency, married/having a partner, living with someone, paid job, income).

EQ-5D-5L index, EQ VAS, ICECAP-A/-O and HAQ-DI scores were calculated for response options of each MSK-HQ items and differences were compared to determine discriminant validity.

#### Reliability

Internal consistency was examined by Cronbach’s alpha (0.7–0.8: acceptable, 0.8–0.9: good > 0.9: excellent) [37]. To assess test–retest reliability in the subsample of 50 participants who have completed the MSK-HQ repeatedly, intraclass correlation coefficient (ICC) was calculated. The ICC can range between 0 (no agreement) and 1 (perfect agreement), indicating the level of agreement (< 0.5 –’poor’, 0.50—0.749 –’moderate’, 0.75—0.900 –’good’, > 0.90 –’excellent’) [38].

Significance level for all tests was accepted as *p* < 0.05. Response option of ʻI do not know / I do not want to answer’ was provided for some items (paid work, income level, MEHM, happinness, EHIS and health care utilisation questions). Respondents who indicated this answer were excluded from the respective analysis of that specific item but their share is provided as ʻnot reported’ among the results. The analysis was performed using IBM SPSS version 25.0 software (IBM Corp., Armonk, NY, USA).

## Results

### Sample characteristics

**Fig. 1:**
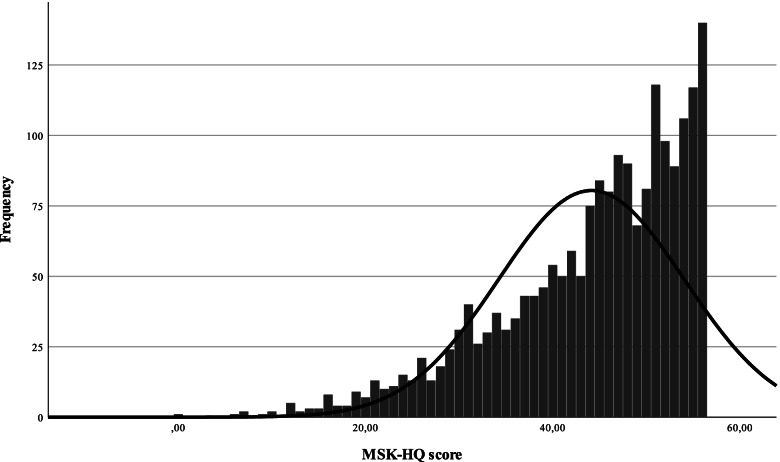
Distribution of MSK-HQ scores

Mean (SD) age of the total sample (*N* = 2004) was 48.3 (16.6) years, 1064 (53.1%) respondents were female. Basic socio-demographic characteristics of the sample is presented in Table 1. The mean (SD) MSK-HQ score was 44.13 (9.94), the distribution in the total sample is presented in Fig. 1.

**Table 1:** Sample characteristics and MSK-HQ scores by socio-demographic groups and self-reported health status categories

Variables	N	%	MSK-HQ score Mean (SD)
**Total**	2004	100.0	44.13 (9.94)
**Gender**			*p* = .001
*Men*	940	46.9	44.85 (9.81)
*Women*	1064	53.1	43.50 (10.01)
**Age**^a^			*p* = .000
*18–24*	192	9.6	47.28 (7.71)
*25–34*	306	15.3	46.26 (8.29)
*35–44*	387	19.3	45.90 (8.95)
*45–54*	332	16.6	43.77 (10.54)
*55–64*	328	16.4	40.54 (10.87)
*65–74*	400	20.0	42.97 (10.42)
*75* +	59	2.9	41.17 (10.15)
**Education**			*p* = .000
*Primary*	608	30.3	42.12 (11.13)
*Secondary*	968	48.3	44.68 (9.34)
*Tertiary*	428	21.4	45.77 (8.97)
**Settlement type**			*p* = .000
*Capital*	358	17.9	45.89 (8.88)
*Town*	1053	52.6	44.26 (9.98)
*Village*	593	29.6	42.86 (10.29)
**Married/having a partner**			*p* = .857
*Yes*	1300	64.9	44.18 (9.74)
*No*	704	35.1	44.04 (10.30)
**Living with someone in the household**			*p* = .224
*Yes*	1686	84.1	44.24 (9.93)
*No*	318	15.9	43.59 (10.00)
**Paid work**			*p* = .000
*Yes*	1158	57.8	45.14 (9.21)
*No*	846	42.2	42.76 (10.71)
**Houshold income category (not reported *N* = 304)**^b^			*p* = .000
*0–718 EUR*	752	44.2	41.78 (10.95)
*718–1435 EUR*	763	44.9	44.76 (9.23)
*Above 1435 EUR*	185	10.9	48.25 (7.27)
**Self-perceived health**			*p* = .000
*Very good*	207	10.3	51.25 (6.25)
*Good*	809	40.4	48.63 (6.20)
*Fair*	784	39.1	41.65 (8.54)
*Bad*	186	9.3	29.38 (9.59)
*Very bad*	18	0.9	20.89 (12.51)
**Long standing illness (not reported *N* = 90)**			*p* = .000
*No*	899	47.0	48.55 (7.10)
*Yes*	1015	53.0	40.26 (10.63)
**GALI (not reported *N* = 32)**			*p* = .000
*Severly limited*	106	5.4	26.75 (11.37)
*Limited, but not severly*	590	29.9	38.34 (9.17)
*Not limited*	1276	64.7	48.26 (6.79)
**Informal care recipient due to MSK problems in the past three months (not reported *N* = 114)**			*p* = .000
*Yes*	113	6.0	28.41 (9.88)
*No*	1777	94.0	45.45 (8.87)
**Informal caregiver**			*p* = .000
*Yes*	238	11.9	41.60 (10.69)
*No*	1766	88.1	44.48 (9.79)

Differences of means were tested by Kruskal–Wallis test and Mann–Whitney U test

*GALI* Global Activity Limitations

^a^There were 55 and 4 participants in age-groups 75–84 and 85–94, respectively

^b^Conversion: 1 EUR = 348.35 HUF (the exchange rate in May, 2020)

Results on the main outcome measures were as follows (mean, SD): EQ-5D-5L index 0.88 (0.20), EQ VAS 75.8 (20.1), HAQ-DI 0.28 (0.47), ICECAP-A 0.77 (0.19), ICECAP-O 0.83 (0.13), WHO-5 57 (21.5) and Happiness VAS 6.44 (2.30). Results of sample summary statistics are summarised in Additional file 1.

### Clinical validity

Statistically significant differences were found in mean MSK-HQ scores by gender (lower score in women) and age groups (lower score for older participants). Furthermore, higher educational level, living in the capital, having paid work and higher household income, as well as being in a better self-percieved general health status were also related to significantly higher MSK-HQ mean score. No significant differences were observed by marital status and if the respondent was living alone or with someone in the household. Both informal caregiver and recipient subgroups had significantly lower mean MSK-HQ score. MSK-HQ scores by socio-demographic subgroups and health status are presented in Table 1.

MSK-HQ scores were significantly lower if respondents had more serious problems on EQ-5D-5L mobility domain and had difficulties with walking. Differences between subgroups with or without MSK diagnoses were also found to be significant. Furthermore, respondents who had used health care services due to their MSK problems 3 months (outpatient visit) or 1 year (inpatient care) prior to the completion of the survey, had significantly lower scores compared to those who had not. MSK-HQ scores by self-reported MSK health status are presented in Table 2.

**Table 2:** MSK-HQ scores by musculoskeletal health problems

Variables	N	%	Mean (SD)
**EQ-5D-5L: Mobility**			*p* = .000
*No*	1254	62.6	49.06 (6.14)
*Slight problems*	414	20.7	41.11 (6.61)
*Moderate problems*	250	12.5	31.23 (8.12)
*Severe problems*	77	3.8	25.19 (7.89)
*Unable to walk*	9	0.4	17.89 (14.82)
**Difficulty in walking 500 m on level** **ground without the use of any aid**^a^** (not reported *N* = 18)**			*p* = .000
*No difficulty*	1527	76.9	47.49 (7.18)
*Any difficulty*	459	23.1	33.05 (9.78)
**Difficulty in walking up or down 12 steps**^a^** (not reported *N* = 13)**			*p* = .000
*No difficulty*	1406	70.6	48.21 (6.58)
*Any difficulty*	585	29.4	34.46 (8.37)
**Low back pain or chronic problem**^a^** (not reported *N* = 146)**			*p* = .000
*No*	1108	59.6	48.25 (7.72)
*Yes, but not diagnosed*	444	23.9	41.73 (8.51)
*Diagnosed*	98	5.3	39.10 (9.95)
*Diagnosed and taking medicine*	208	11.2	32.51 (10.56)
**Back pain or chronic problem**^a^ **(not reported *N* = 110)**			*p* = .000
*No*	1342	70.9	47.14 (8.24)
*Yes, but not diagnosed*	277	14.6	41.43 (8.61)
*Diagnosed*	103	5.4	38.87 (9.62)
*Diagnosed and taking medicine*	172	9.1	30.48 (10.20)
**Neck pain or chronic problem**^a^ **(not reported *N* = 106)**			*p* = .000
*No*	1389	73.2	46.85 (8.44)
*Yes, but not diagnosed*	310	16.3	41.14 (8.91)
*Diagnosed*	78	4.1	36.15 (10.81)
*Diagnosed and taking medicine*	121	6.4	30.00 (9.87)
**Osteoarthritis of the knee**^a^ **(not reported *N* = 101)**			*p* = .000
*No*	1468	77.1	46.49 (8.67)
*Yes, but not diagnosed*	164	8.6	40.68 (9.13)
*Diagnosed*	114	6.0	39.77 (9.53)
*Diagnosed and taking medicine*	157	8.3	32.11 (10.36)
**Osteoarthritis of the hip**^a^ **(not reported *N* = 98)**			*p* = .000
*No*	1602	84.1	46.20 (8.58)
*Yes, but not diagnosed*	81	4.2	38.95 (11.17)
*Diagnosed*	93	4.9	36.85 (10.48)
*Diagnosed and taking medicine*	130	6.8	30.97 (10.00)
**Hospital admission in the last 1 year due to MSK health problems (not reported *N* = 39)**			*p* = .000
*Yes*	235	12.0	36.06 (11.48)
*No*	1730	88.0	45.35 (9.12)
**Seen by specialist in the last 3 months due to MSK health problems (not reported *N* = 42)**			*p* = .000
*Yes*	322	16.4	36.63 (11.33)
*No*	1640	83.6	45.79 (8.84)
**Seen by family doctor in the last 3 months due to MSK health problems (not reported *N* = 34)**			*p* = .000
*Yes*	231	11.7	34.87 (10.65)
*No*	1739	88.3	45.53 (9.02)

Differences of means were tested by Kruskal–Wallis test

^a^Questions of the European Health Interview Survey (EHIS) were applied

### Convergent validity

The strongest correlation was observed between MSK-HQ scores and EQ-5D-5L index values, followed by HAQ-DI and EQ VAS (the negative correlation with the HAQ-DI is due to the reverse scoring of the measure). MSK-HQ correlated moderately with ICECAP-A/-O and WHO-5, observed coefficients were fairly similar. However, the correlation with Happiness VAS was only weak. All correlations were found to be significant. Results are presented in Table 3.

**Table 3:** Spearman’s correlations of MSK-HQ scores with the EQ-5D-5L index, EQ VAS, HAQ-DI, WHO-5, Happiness VAS, ICECAP-A (age group 18–64) and ICECAP-O (age group 65 +) scores

	MSK-HQ	EQ-5D-5L index score	EQ VAS	HAQ-DI	ICECAP-A	ICECAP-O	WHO-5 score	Happiness VAS
MSK-HQ	1.000	.788	.644	-.698	.471	.460	.443	.317
EQ-5D-5L index score		1.000	.661	-.702	.566	.474	.485	.388
EQ VAS			1.000	-.550	.517	.501	.517	.450
HAQ-DI				1.000	-.379	-.427	-.324	-.241
ICECAP-A					1.000	–	.628	.606
ICECAP-O						1.000	.606	.579
WHO-5 score							1.000	.621
Happiness VAS								1.000

All correlations are significant at the 0.01 level (2-tailed)

In most cases, the association of MSK-HQ items with EQ-5D-5L domains and index scores were strong or moderate, with some exceptions. Weak correlations were observed for ʻunderstanding condition’ item of MSK-HQ in all instances. Furthermore, ʻanxiety/depression’ and ʻself-care’ domains of EQ-5D-5L were also found to be weakly associated with some of the MSK-HQ items. All correlations were significant. Detailed results are presented in Additional file 2.

HAQ-DI domains showed moderate or strong correlations with most of the MSK-HQ items, except with ʻunderstanding condition’ which correlated weakly with all the eight HAQ-DI items and the HAQ-DI score, and with six further items. All correlations were found to be significant. Results are summarized in Additional file 3.

### Discriminant validity

Respondents with more severe MSK problems indicated worse states on the outcome measures with some exceptions. Among respondents who reported no problems at all for ʻunderstanding condition’ (*n* = 242) and ʻconfidence in managing’ (*n* = 107) MSK-HQ items, a slight increase (better status) was observed in the scores of all scales. Differences between subgroups were found to be significant for all MSK-HQ items and measurement scales. Results are shown in Additional file 4.

### Test–retest reliability and internal consistency

All the 50 respondents of the retest subsample answered each question. Among them, the mean MSK-HQ score was 41.30 (± 13.79) on the first, and 42.92 (± 13.53) on the second round. The ICC was 0.936 (95% CI: 0.884 – 0.964), indicating excellent level of agreement. Cronbach’s alpha was 0.924, indicating good internal consistency.

### Regression

Results of the regression analysis are shown in Additional file 5. In models 1 and 2, the EQ-5D-5L and the HAQ-DI were significantly associated with MSK-HQ scores (marginal effects 38.39 and -14.98, respectively). Model 1, which included the sociodemographic background variables and EQ-5D-5L, explained 63% of variance in MSK-HQ score. The performance decreased to 54% for model 2, in which HAQ-DI was included instead of EQ-5D-5L. In model 3, when the two scales were included together, their influence on MSK-HQ remained significant, but with lower marginal effects (27.16 and -6.47, respectively). The best model performance was observed for model 3 which could explain 66% of the variance in MSK-HQ score (*R*^2^ = 0.663). For control variables in model 3, significant positive associations were detected with type of residency (living in the capital) and income (above 1435 EUR). Being a women, higher age, primary education and having a partner were found to be negatively associated with MSK-HQ score.

## Discussion

We have produced the Hungarian language version of the MSK-HQ measure and tested its psychometric performance in an online cross-sectional survey among the general adult population of Hungary. The tool has been proven to have excellent measurement properties in terms of construct validity, internal consistency, and test–retest reliability. To date, it has been the first MSK-HQ study that was carried out on a representative sample of the general adult population, involving a relatively high sample size compared to previous studies [8, 9, 11, 12, 14, 15, 17, 18]. Furthermore, this has been the first study to assess the association of the MSK-HQ score with EQ-5D-5L and HAQ-DI in a population sample. Another strength of this research is that a number of validated scales were applied to capture aspects of well-being alongside the MSK-HQ and their associations were investigated. Using the questions of the Eurostat (EHIS) to assess citizens’ self-reported MSK problems and diagnoses provides the first basis for investigating the link between routinely collected national statistics and MSK-HQ scores in further studies.

The new language version of MSK-HQ performed well in our sample as it could differentiate between both socio-demographic groups and self-reported health status categories that were expected to differ in their MSK health. The results of the OLS regression analyses also revealed that socio-demographic characteristics, such as gender, age, educational level, marital status, place of residency and income level are significant determinants of MSK-HQ score. Subgroup comparison showed that respondents with self-reported MSK disorders, walking disabilities (based on EHIS questions), prior use of health care services or informal care due to MSK problems had significantly lower MSK-HQ score compared to those who did not report such problems. It can be further concluded that participants with more severe complaints had worse MSK-HQ score. Similar subgroup comparisons have not been published before, therefore our results contribute to the literature by filling this gap.

Our results have further confirmed the strong correlation of MSK-HQ with EQ-5D-5L index, EQ VAS and HAQ-DI that was described in previous studies. In our analysis, we observed a coefficient of 0.788 between MSK-HQ and EQ-5D-5L, which is comparable with results from studies involving MSK patient samples [8, 9, 11, 15]. We would like to emphasise that in our analysis the country-specific Hungarian tariffs were used to calculate the EQ-5D-5L index scores, while at the time of former publications only the value set for England was available [28, 39]. Strong correlation between MSK-HQ and HAQ-DI was observed, with a coefficient of -0.698, although it was lower compared to what has been published in inflammatory arthritis by Norton et al. (*r* = 0.81) [9].

Well-being measurement scales are not commonly used as reference standards, resulting in a scarcity of data within the field of MSK disorders. We found it relevant to broaden the scope and include the WHO-5, ICECAP-A/O and Happiness VAS. Our results have confirmed that the correlation of MSK-HQ with these tools are weaker (but still moderate and significant) compared to that seen with HRQoL (EQ-5D-5L) and physical functioning (HAQ-DI) measures.

In previous studies, ʻunderstanding condition’ and ʻconfidence in managing’ had weaker relationship with MSK-HQ total score compared to other items [8, 9]. The results of our study further reinforce these observations as we found that ʻunderstanding condition’ weakly correlated with all EQ-5D-5L domains. For ʻconfidence in managing’, the correlation was moderate with most of the domains, although coefficients were apparently lower compared to that seen with other items. The same trend was observed for both items in the comparison with HAQ-DI domains.

In our study the Cronbach’s alpha of the Hungarian version of MSK-HQ was 0.924, which confirms the good internal consistency of the measure [8, 9, 11, 14, 15]. In addition, the scale was proved to be reliable with an ICC of 0.936, similar to that seen with the Arabic, Turkish and Italian version, although still higher compared to the English, Danish, German, and Norvegian versions [8, 9, 11, 14, 15, 18, 40]. The short time interval between the two assessments partly explains our higher ICC results.

The multiple regression analysis revealed that both EQ-5D-5L and HAQ-DI are significant determinants of MSK-HQ score. The multiple regression analysis also confirmed that MSK-HQ score is significantly assoicated with general HRQoL and phsical functional status. Similar results have been published by Burgess et al., who examined the predictive ability of MSK case-mix adjustment models and found EQ-5D-5L to be significant predictor of 6 months MSK-HQ score [41]. Furthermore, important sociodemographic factors were identified as being significant determinants of MSK-HQ score, such as gender, age, monthly income and educational level. The best predictive ability was observed if both EQ-5D-5L and HAQ-DI were included in the regression analysis along with sociodemographic variables.

Our study has some limitations that must be addressed. Due to the cross-sectional design of our study, we could not assess responsiveness and the minimal important change (MIC). Although, considering previously published MIC results, the results observed by MSK health problems suggest clinically meaningful differences [13, 15, 17]. Inconsistent results were observed for ʻunderstanding condition’ and ʻconfidence in managing’ items as an increase was shown in the values of reference scales among those who reported the worst level problems. We further investigated these cases and found that most of these respondents had had no MSK health problems in the last 2 weeks before the completion of the survey, thus these questions were probably not really relevant for them. This issue deserves further consideration (and maybe alternative scoring) for the use of MSK-HQ in non-clinical samples. The survey was conducted online which may cause selection bias. Therefore, caution is needed when using our MSK-HQ results as population norms. Despite the wealth of information acquired from this research, data on effect size, smallest detectable change and MIC are lacking and careful evaluation involving predefined MSK patient groups is still needed. Furthermore, the short time interval between test and retest measures could be a possible source of bias regarding ICC results. However, in previous studies the MSK-HQ was found to have excellent reliability even with lower ICC values and with greater time intervals between the two measures, indicating that these uncertainties probably have an insignificant effect on our conclusion on the reliability of the Hungarian version of the scale.

## Conclusions

In summary, we successfully performed the psychometric validation of the Hungarian version MSK-HQ, thus a new language version of the MSK-HQ is now available in Europe. Our study has confirmed the validity of the tool on a representative population sample that broadens the applicability of the MSK-HQ measure. The strong association between MSK-HQ and EQ-5D-5L and HAQ-DI observed in MSK patients has been confimed in this much broader setting. Socio-demographic characteristics have been proved to be significant determinants of MSK-HQ score, which deserves consideration in clinical studies. Moderate but significant associations of MSK-HQ with ICECAP-A/-O, WHO-5 and Happiness VAS measures draws attention to the impact of MSK problems on well-being. The results of our study can be utilized and implemented by clinicians and policy makers as well. The appropriate and aggregated use of MSK-HQ could help to proactively explore MSK health problems of individuals outside the healthcare system, support medical decision making and contribute to develop health policies aiming to improve the HRQoL of patients with MSK disorders.

## Supplementary Information

**Additional file 1.** Sample summary statistics for MSK-HQ, EQ-5D-5L, EQ VAS, HAQ-DI, ICECAP-A/O, WHO-5 and Happiness VAS.

**Additional file 2.** Spearman correlations of MSK-HQ items with the EQ-5D-5L domains.

**Additional file 3.** Spearman correlations of MSK-HQ items with the HAQ-DI domains.

**Additional file 4.** EQ-5D-5L index, EQ VAS, ICECAP-A/O and HAQ-DI scores of respondents on each level of MSK-HQ domains.

**Additional file 5.** Results of the regression analysis (t-statistics in parentheses).

## Data Availability

The data that support the findings of this study are available from the corresponding author upon reasonable request.

## Abbreviations

COSMIN: COnsensus-based Standards for the selection of health status Measurement INstruments
EHIS: European Health Interview Survey
HAQ-DI: Health Assessment Questionnaire – Disability Index
HRQoL: Health-Related Quality of Life
ICC: Intraclass correlation coefficient
ICECAP-A: ICEpop CAPability measure for Adults
ICECAP-O: ICEpop CAPability measure for Older people
MEHM: Minimum European Health Module
MSK: Musculoskeletal
MSK-HQ: Musculoskeletal Health Questionnaire
OLS: Ordinary Least Square
SD: Standard Deviation
VAS: Visual analog scale
WHO-5: World Health Organisation-Five Well-Being Index
