# Supplementary material for: Validation of the Musculoskeletal Health Questionnaire in a general population sample: a cross-sectional online survey in Hungary
Source: BMC Musculoskelet Disord. 2022 Aug 13;23:771. doi: 10.1186/s12891-022-05716-9 (PMC9375429; doi:10.1186/s12891-022-05716-9)
Supplement: Supplementary file 1 — Additional file 1. Sample summary statistics for MSK-HQ, EQ-5D-5L, EQ VAS, HAQ-DI, ICECAP-A/O, WHO-5 and Happiness VAS. [file 12891_2022_5716_MOESM1_ESM.docx]

**Additional file 1: Sample summary statistics for MSK-HQ, EQ-5D-5L, EQ VAS, HAQ-DI, ICECAP-A/O, WHO-5 and Happiness VAS**

|  | **Total sample** | | | **Age group 18-64** | | | **Age-group 65+** | | |
| --- | --- | --- | --- | --- | --- | --- | --- | --- | --- |
|  | **N** | **Mean** | **SD** | **N** | **Mean** | **SD** | **N** | **Mean** | **SD** |
| **MSK-HQ (0-56)** | 2004 | 44.13 | 9.94 | 1545 | 44.55 | 9.76 | 459 | 42.74 | 10.39 |
| **EQ-5D-5L index^a^ ((-0.848)-1)** | 2004 | 0.88 | 0.20 | 1545 | 0.89 | 0.19 | 459 | 0.85 | 0.20 |
| **EQ VAS (0-100)** | 2004 | 75.81 | 20.10 | 1545 | 76.64 | 19.96 | 459 | 73.05 | 20.34 |
| **HAQ-DI (0-3)** | 2004 | 0.28 | 0.47 | 1545 | 0.24 | 0.45 | 459 | 0.39 | 0.50 |
| **ICECAP-A^b^ (0-1)** | 1545 | 0.77 | 0.19 | 1545 | 0.77 | 0.19 | NA | NA | NA |
| **ICECAP-O^b^ (0-1)** | 459 | 0.83 | 0.13 | NA | NA | NA | 459 | 0.83 | 0.13 |
| **WHO-5 score (0-100)** | 2004 | 57.00 | 21.49 | 1545 | 56.20 | 21.32 | 459 | 59.70 | 21.86 |
| **Happiness VAS (0-10) (not reported: 19)** | 1985 | 6.44 | 2.30 | 1532 | 6.43 | 2.34 | 453 | 6.49 | 2.15 |

^a^ Calculated with value set for Hungary

**^b^**ICECAP-A and ICECAP-O measures were applied in age groups 18-64 and 65 and over, respectively. ICECAP-/-O index scores were calculated with value set for the UK.
