# Supplementary material for: Validation of the Musculoskeletal Health Questionnaire in a general population sample: a cross-sectional online survey in Hungary
Source: BMC Musculoskelet Disord. 2022 Aug 13;23:771. doi: 10.1186/s12891-022-05716-9 (PMC9375429; doi:10.1186/s12891-022-05716-9)
Supplement: Supplementary file 2 — Additional file 2. Spearman correlations of MSK-HQ items with the EQ-5D-5L domains. [file 12891_2022_5716_MOESM2_ESM.docx]

**Additional file 2: Spearman correlations of MSK-HQ items with the EQ-5D-5L domains**

|  | **Mobility** | **Self-care** | **Usual activities** | **Pain / discomfort** | **Anxiety / depression** | **EQ-5D-5L index** |
| --- | --- | --- | --- | --- | --- | --- |
| 1. **Pain/stiffness during the day** | -0.566 | -0.397 | -0.579 | -0.661 | -0.297 | 0.649 |
| 1. **Pain/stiffness at night** | -0.511 | -0.399 | -0.524 | -0.586 | -0.314 | 0.584 |
| 1. **Walking** | -0.775 | -0.502 | -0.629 | -0.582 | -0.278 | 0.664 |
| 1. **Washing/dressing** | -0.588 | -0.647 | -0.604 | -0.518 | -0.268 | 0.581 |
| 1. **Physical activity levels** | -0.682 | -0.480 | -0.634 | -0.636 | -0.305 | 0.686 |
| 1. **Work/daily routine** | -0.648 | -0.493 | -0.673 | -0.643 | -0.333 | 0.691 |
| 1. **Social activities and hobbies** | -0.635 | -0.510 | -0.637 | -0.587 | -0.352 | 0.654 |
| 1. **Needing help** | -0.562 | -0.566 | -0.597 | -0.492 | -0.303 | 0.553 |
| 1. **Sleep** | -0.496 | -0.434 | -0.521 | -0.561 | -0.341 | 0.577 |
| 1. **Fatigue or low energy** | -0.407 | -0.297 | -0.441 | -0.547 | -0.446 | 0.590 |
| 1. **Emotional well-being** | -0.464 | -0.406 | -0.495 | -0.568 | -0.500 | 0.615 |
| 1. **Understanding condition** | -0.163 | -0.095 | -0.155 | -0.229 | -0.197 | 0.244 |
| 1. **Confidence in managing** | -0.389 | -0.291 | -0.414 | -0.454 | -0.306 | 0.486 |
| 1. **Overall impact** | -0.616 | -0.453 | -0.615 | -0.688 | -0.360 | 0.706 |

All correlations are significant at the p<0.01 level
