# Supplementary material for: Validation of the Musculoskeletal Health Questionnaire in a general population sample: a cross-sectional online survey in Hungary
Source: BMC Musculoskelet Disord. 2022 Aug 13;23:771. doi: 10.1186/s12891-022-05716-9 (PMC9375429; doi:10.1186/s12891-022-05716-9)
Supplement: Supplementary file 3 — Additional file 3. Spearman correlations of MSK-HQ items with the HAQ-DI domains. [file 12891_2022_5716_MOESM3_ESM.docx]

**Additional file 3: Spearman correlations of MSK-HQ items with the HAQ-DI domains**

|  | **Dressing / grooming** | **Arising** | **Eating** | **Walking** | **Hygiene** | **Reach** | **Grip** | **Activities** | **HAQ-DI index** |
| --- | --- | --- | --- | --- | --- | --- | --- | --- | --- |
| 1. **Pain/stiffness during the day** | -0.426 | -0.510 | -0.273 | -0.486 | -0.451 | -0.498 | -0.305 | -0.516 | -0.591 |
| 1. **Pain/stiffness at night** | -0.421 | -0.482 | -0.286 | -0.456 | -0.426 | -0.456 | -0.319 | -0.459 | -0.530 |
| 1. **Walking** | -0.527 | -0.609 | -0.327 | -0.637 | -0.573 | -0.537 | -0.324 | -0.603 | -0.667 |
| 1. **Washing/dressing** | -0.617 | -0.548 | -0.367 | -0.563 | -0.590 | -0.574 | -0.372 | -0.582 | -0.633 |
| 1. **Physical activity levels** | -0.506 | -0.579 | -0.308 | -0.594 | -0.529 | -0.541 | -0.311 | -0.591 | -0.657 |
| 1. **Work/daily routine** | -0.517 | -0.573 | -0.351 | -0.587 | -0.534 | -0.569 | -0.353 | -0.615 | -0.681 |
| 1. **Social activities and hobbies** | -0.518 | -0.549 | -0.357 | -0.583 | -0.513 | -0.526 | -0.374 | -0.564 | -0.619 |
| 1. **Needing help** | -0.545 | -0.540 | -0.406 | -0.563 | -0.538 | -0.550 | -0.450 | -0.551 | -0.588 |
| 1. **Sleep** | -0.441 | -0.501 | -0.308 | -0.468 | -0.434 | -0.469 | -0.348 | -0.474 | -0.539 |
| 1. **Fatigue or low energy** | -0.334 | -0.390 | -0.243 | -0.386 | -0.319 | -0.381 | -0.265 | -0.394 | -0.453 |
| 1. **Emotional well-being** | -0.400 | -0.437 | -0.303 | -0.442 | -0.406 | -0.441 | -0.339 | -0.456 | -0.505 |
| 1. **Understanding condition** | -0.094 | -0.133 | -0.055 | -0.131 | -0.075 | -0.112 | -0.101 | -0.114 | -0.158 |
| 1. **Confidence in managing** | -0.289 | -0.351 | -0.208 | -0.328 | -0.301 | -0.343 | -0.209 | -0.340 | -0.403 |
| 1. **Overall impact** | -0.472 | -0.534 | -0.327 | -0.521 | -0.481 | -0.513 | -0.325 | -0.548 | -0.624 |

All correlations are significant at the 0.01 level, except the correlation between „Eating” and „Understanding condition” (p=0.014)
