# Supplementary material for: Validation of the Musculoskeletal Health Questionnaire in a general population sample: a cross-sectional online survey in Hungary
Source: BMC Musculoskelet Disord. 2022 Aug 13;23:771. doi: 10.1186/s12891-022-05716-9 (PMC9375429; doi:10.1186/s12891-022-05716-9)
Supplement: Supplementary file 4 — Additional file 4. EQ-5D-5L index, EQ VAS, ICECAP-A/O and HAQ-DI scores of respondents on each level of MSK-HQ domains. [file 12891_2022_5716_MOESM4_ESM.docx]

**Additional file 4: EQ-5D-5L index, EQ VAS, ICECAP-A/O and HAQ-DI scores of respondents on each level of MSK-HQ domains**

|  | **N (%)** | **EQ-5D-5L index**  **((-0.848)-1)** | **EQ VAS (0-100)** | **HAQ-DI (0-3)** | **ICECAP-A^a^ (0-1)**  **N=1545** | **ICECAP-O^a^ (0-1)**  **N=459** |
| --- | --- | --- | --- | --- | --- | --- |
| **MSK-HQ total (0-56)** | 2004 (100) | 0.88 | 75.81 | 0.28 | 0.77 | 0.83 |
| 1. **Pain/stiffness during the day** |  | p= .000 | p= .000 | p= .000 | p= .000 | p= .000 |
| *Not at all* | 697 (34.8) | 0.97 | 86.13 | 0.05 | 0.84 | 0.88 |
| *Slightly* | 676 (33.7) | 0.91 | 77.78 | 0.20 | 0.77 | 0.84 |
| *Moderately* | 516 (25.7) | 0.79 | 65.55 | 0.49 | 0.70 | 0.79 |
| *Fairly severe* | 106 (5.3) | 0.54 | 50.02 | 1.09 | 0.65 | 0.76 |
| *Very severe* | 9 (0.4) | -0.10 | 21.44 | 1.54 | 0.45 | 0.69 |
| 1. **Pain/stiffness at night** |  | p= .000 | p= .000 | p= .000 | p= .000 | p= .000 |
| *Not at all* | 1122 (56.0) | 0.95 | 82.99 | 0.10 | 0.82 | 0.86 |
| *Slightly* | 547 (27.3) | 0.86 | 72.12 | 0.35 | 0.73 | 0.82 |
| *Moderately* | 264 (13.2) | 0.74 | 61.56 | 0.59 | 0.69 | 0.79 |
| *Fairly severe* | 61 (3.0) | 0.46 | 44.62 | 1.27 | 0.60 | 0.69 |
| *Very severe* | 10 (0.5) | 0.07 | 34.30 | 1.51 | 0.50 | 0.78 |
| 1. **Walking** |  | p= .000 | p= .000 | p= .000 | p= .000 | p= .000 |
| Not at all | 1161 (57.9) | 0.96 | 83.35 | 0.07 | 0.82 | 0.86 |
| Slightly | 484 (24.2) | 0.87 | 72.31 | 0.33 | 0.74 | 0.83 |
| Moderately | 212 (10.6) | 0.75 | 61.63 | 0.69 | 0.70 | 0.81 |
| Severly | 138 (6.9) | 0.49 | 49.50 | 1.10 | 0.61 | 0.73 |
| Unable to walk | 9 (0.4) | -0.14 | 29.33 | 2.17 | 0.35 | 0.67 |
| 1. **Washing/dressing** |  | p= .000 | p= .000 | p= .000 | p= .000 | p= .000 |
| *Not at all* | 1489 (74.3) | 0.94 | 81.29 | 0.11 | 0.80 | 0.85 |
| *Slightly* | 345 (17.2) | 0.78 | 64.96 | 0.58 | 0.70 | 0.81 |
| *Moderately* | 121 (6.0) | 0.60 | 52.56 | 1.05 | 0.64 | 0.74 |
| *Severly* | 43 (2.1) | 0.39 | 44.21 | 1.22 | 0.60 | 0.63 |
| *Unable to wash or dress myself* | 6 (0.3) | -0.11 | 35.83 | 2.38 | 0.47 | 0.76 |
| 1. **Physical activity levels** |  | p= .000 | p= .000 | p= .000 | p= .000 | p= .000 |
| Not at all | 957 (47.8) | 0.97 | 84.70 | 0.05 | 0.82 | 0.88 |
| Slightly | 605 (30.2) | 0.89 | 75.13 | 0.25 | 0.76 | 0.83 |
| Moderately | 270 (13.5) | 0.77 | 62.88 | 0.64 | 0.70 | 0.79 |
| Very much | 150 (7.5) | 0.55 | 51.39 | 0.94 | 0.62 | 0.72 |
| Unable to do physical activities | 22 (1.1) | 0.15 | 33.32 | 1.71 | 0.53 | 0.64 |
| 1. **Work/daily routine** |  | p= .000 | p= .000 | p= .000 | p= .000 | p= .000 |
| *Not at all* | 987 (49.3) | 0.97 | 84.89 | 0.04 | 0.83 | 0.87 |
| *Slightly* | 608 (30.3) | 0.89 | 74.32 | 0.28 | 0.76 | 0.84 |
| *Moderately* | 251 (12.5) | 0.74 | 61.58 | 0.67 | 0.67 | 0.78 |
| *Severly* | 139 (6.9) | 0.52 | 49.38 | 1.06 | 0.62 | 0.72 |
| *Extremely* | 19 (0.9) | 0.14 | 33.47 | 1.55 | 0.49 | 0.67 |
| 1. **Social activities and hobbies** |  | p= .000 | p= .000 | p= .000 | p= .000 | p= .000 |
| *Not at all* | 1277 (63.7) | 0.95 | 83.10 | 0.08 | 0.81 | 0.86 |
| *Slightly* | 432 (21.6) | 0.84 | 69.81 | 0.39 | 0.74 | 0.81 |
| *Moderately* | 178 (8.9) | 0.70 | 57.50 | 0.73 | 0.66 | 0.78 |
| *Severely* | 95 (4.7) | 0.49 | 48.97 | 1.17 | 0.59 | 0.68 |
| *Extremely* | 22 (1.1) | 0.16 | 34.86 | 1.55 | 0.54 | 0.67 |
| 1. **Needing help** |  | p= .000 | p= .000 | p= .000 | p= .000 | p= .000 |
| *Not at all* | 1551 (77.4) | 0.94 | 80.78 | 0.12 | 0.80 | 0.85 |
| *Rarely* | 241 (12.0) | 0.80 | 66.56 | 0.54 | 0.73 | 0.82 |
| *Sometimes* | 133 (6.6) | 0.65 | 53.91 | 0.97 | 0.64 | 0.74 |
| *Frequently* | 64 (3.2) | 0.40 | 44.83 | 1.25 | 0.57 | 0.65 |
| *All the time* | 15 (0.7) | 0.09 | 37.07 | 1.78 | 0.52 | 0.68 |
| 1. **Sleep** |  | p= .000 | p= .000 | p= .000 | p= .000 | p= .000 |
| *Not at all* | 1135 (56.6) | 0.95 | 83.13 | 0.10 | 0.82 | 0.87 |
| *Rarely* | 444 (22.2) | 0.87 | 72.09 | 0.30 | 0.74 | 0.82 |
| *Sometimes* | 204 (10.2) | 0.77 | 66.36 | 0.55 | 0.73 | 0.78 |
| *Frequently* | 174 (8.7) | 0.65 | 56.28 | 0.84 | 0.66 | 0.72 |
| *Every night* | 47 (2.3) | 0.47 | 47.68 | 1.09 | 0.58 | 0.72 |
| 1. **Fatigue or low energy** |  | p= .000 | p= .000 | p= .000 | p= .000 | p= .000 |
| *Not at all* | 451 (22.5) | 0.97 | 87.41 | 0.06 | 0.88 | 0.89 |
| *Slight* | 765 (38.2) | 0.93 | 81.09 | 0.18 | 0.81 | 0.85 |
| *Moderate* | 440 (22.0) | 0.83 | 69.34 | 0.39 | 0.74 | 0.79 |
| *Severe* | 291 (14.5) | 0.72 | 59.05 | 0.62 | 0.63 | 0.71 |
| *Extreme* | 57 (2.8) | 0.57 | 48.77 | 0.70 | 0.51 | 0.51 |
| 1. **Emotional well-being** |  | p= .000 | p= .000 | p= .000 | p= .000 | p= .000 |
| *Not at all* | 1062 (53.0) | 0.96 | 84.15 | 0.10 | 0.84 | 0.87 |
| *Slightly* | 529 (26.4) | 0.88 | 73.87 | 0.30 | 0.76 | 0.84 |
| *Moderately* | 250 (12.5) | 0.75 | 61.60 | 0.58 | 0.68 | 0.73 |
| *Severely* | 137 (6.8) | 0.59 | 51.68 | 0.84 | 0.55 | 0.64 |
| *Extremely* | 26 (1.3) | 0.30 | 38.54 | 1.19 | 0.48 | 0.66 |
| 1. **Understanding condition** |  | p= .000 | p= .000 | p= .000 | p= .000 | p= .000 |
| *Completely* | 865 (43.2) | 0.92 | 82.03 | 0.19 | 0.82 | 0.85 |
| *Very well* | 317 (15.8) | 0.85 | 73.32 | 0.35 | 0.75 | 0.83 |
| *Moderately* | 408 (20.4) | 0.82 | 67.98 | 0.43 | 0.73 | 0.78 |
| *Slightly* | 172 (8.6) | 0.81 | 67.82 | 0.37 | 0.68 | 0.79 |
| *Not at all* | 242 (12.1) | 0.91 | 75.75 | 0.17 | 0.76 | 0.84 |
| 1. **Confidence in managing** |  | p= .000 | p= .000 | p= .000 | p= .000 | p= .000 |
| *Extremely* | 535 (26.7) | 0.96 | 86.26 | 0.07 | 0.85 | 0.90 |
| *Very* | 698 (34.8) | 0.92 | 80.41 | 0.21 | 0.81 | 0.86 |
| *Moderately* | 508 (25.3) | 0.79 | 65.06 | 0.46 | 0.70 | 0.76 |
| *Slightly* | 156 (7.8) | 0.74 | 61.34 | 0.52 | 0.65 | 0.76 |
| *Not at all* | 107 (5.3) | 0.75 | 65.74 | 0.49 | 0.70 | 0.76 |
| 1. **Overall impact** |  | p= .000 | p= .000 | p= .000 | p= .000 | p= .000 |
| *Not at all* | 760 (37.9) | 0.98 | 86.32 | 0.03 | 0.84 | 0.88 |
| *Slightly* | 685 (34.2) | 0.91 | 77.53 | 0.22 | 0.78 | 0.83 |
| *Moderately* | 363 (18.1) | 0.79 | 63.56 | 0.52 | 0.70 | 0.81 |
| *Very much* | 158 (7.9) | 0.59 | 53.91 | 0.92 | 0.64 | 0.72 |
| *Extremely* | 38 (1.9) | 0.32 | 42.74 | 1.17 | 0.57 | 0.54 |

Differences of means were tested by Kruskal-Wallis test.
