# Supplementary material for: Validation of the Musculoskeletal Health Questionnaire in a general population sample: a cross-sectional online survey in Hungary
Source: BMC Musculoskelet Disord. 2022 Aug 13;23:771. doi: 10.1186/s12891-022-05716-9 (PMC9375429; doi:10.1186/s12891-022-05716-9)
Supplement: Supplementary file 5 — Additional file 5. Results of the regression analysis (t-statistics in parentheses). [file 12891_2022_5716_MOESM5_ESM.docx]

**Additional file 5: Results of the regression analysis (t-statistics in parentheses)**

| **Independent variables** | **Dependent variable: MSK-HQ score** | | |
| --- | --- | --- | --- |
|  | **Model 1** | **Model 2** | **Model 3** |
| Women (reference: men) | -1.523**  (-5.232) | -0.861**  (-2.675) | -1.226**  (-4.426) |
| Age (years) | -0.049**  (-5.191) | -0.007  (-0.690) | -0.021*  (-2.280) |
| Education (reference: tertiary) |  |  |  |
| primary | -1.390**  (-3.450) | -1.274**  (-2.862) | -1.115**  (-2.914) |
| secondary | -0.553  (-1.515) | -0.570  (-1.416) | -0.502  (-1.449) |
| Residency (reference: village) |  |  |  |
| capital | 1.225**  (2.967) | 1.003*  (2.199) | 0.952*  (2.428) |
| town | 0.374  (1.179) | 0.436  (1.248) | 0.281  (0.934) |
| Married/having a partner (reference: no) | -1.049**  (-3.120) | -0.455  (-1.228) | -0.950**  (-2.975) |
| Living with someone (reference: no) | 0.113  (0.251) | -0.334  (-0.673) | 0.164  (0.384) |
| Paid job (reference: no) | -0.329  (-1.098) | -0.027  (-0.081) | -0.321  (-1.131) |
| Income category (reference: 0-718) |  |  |  |
| 718-1435 | 0.233  (0.668) | 0.462  (1.203) | 0.040  (0.120) |
| above 1435 | 1.383*  (2.522) | 1.994**  (3.298) | 1.209*  (2.323) |
| not reported | 0.834  (1.922) | 0.928  (1.936) | 0.546  (1.325) |
| EQ-5D-5L index | 38.389**  (52.648) | - | 27.162**  (26.538) |
| HAQ-DI | - | -14.977**  (-43.799) | -6.473**  (-14.885) |
| Constant | 14.340**  (13.279) | 49.420**  (53.539) | 24.473**  (19.895) |
| Observations  R squared  F > test | 2004  0.625  255.232** | 2004  0.543  182.055** | 2004  0.663  279.096** |

Note: Age is included as a continous variable. Education, residence and income category are included as dummy variables.

**p<0.01; *p<0.05
